# Extracellular Vesicle Delivery of TRAIL Eradicates Resistant Tumor Growth in Combination with CDK Inhibition by Dinaciclib

**DOI:** 10.3390/cancers12051157

**Published:** 2020-05-04

**Authors:** Changhong Ke, Huan Hou, Jiayu Li, Kui Su, Chaohong Huang, Yue Lin, Zhiqiang Lu, Zhiyun Du, Wen Tan, Zhengqiang Yuan

**Affiliations:** 1 Institute of Biomedical and Pharmaceutical Sciences, Guangdong University of Technology, Guangzhou 51006, China; masterchke@163.com (C.K.); HuanHou123@163.com (H.H.); kuisu_dgut@163.com (K.S.); c504496984@163.com (C.H.); Linyrw@163.com (Y.L.); luzq85@163.com (Z.L.); zhiyundu@gdut.edu.cn (Z.D.); went@gdut.edu.cn (W.T.); 2 School of Industrial Design and Ceramic Art of Foshan University, Foshan 528000 China; chaimu@fosu.edu.cn

**Keywords:** extracellular vesicle, TRAIL, dinaciclib, combination therapy, cancer resistance

## Abstract

Tumour necrosis factor (TNF)-related apoptosis inducing ligand (TRAIL) is a promising anti-cancer agent that rapidly induces apoptosis in cancer cells. Unfortunately, the clinical application of recombinant TRAIL (rTRAIL) has been hampered by its common cancer resistance. Naturally TRAIL is delivered as a membrane-bound form by extracellular vesicles (EV-T) and is highly efficient for apoptosis induction. SCH727965 (dinaciclib), a potent cyclin-dependent kinase (CDK) inhibitor, was shown to synergize with other drugs to get better efficacy. However, it has never been investigated if dinaciclib coordinates with EV-T to enhance therapeutic results. This study explores the potential of combination therapy with EV-T and dinaciclib for cancer treatment. EV-T was successfully derived from human TRAIL transduced cells and shown to partially overcome resistance of A549 cells. Dinaciclib was shown to drastically enhance EV-T killing effects on cancer lines that express good levels of death receptor (DR) 5, which are associated with suppression of CDK1, CDK9 and anti-apoptotic proteins. Combination therapy with low doses of EV-T and dinaciclib induced strikingly enhanced apoptosis and led to complete regression in A549 tumors without any adverse side effects observed in a subcutaneous xenograft model. Tumor infiltration of mass NK cells and macrophages was also observed. These observations thus indicate that the combination of EV-T with dinaciclib is a potential novel therapy for highly effective and safe cancer treatment.

## 1. Introduction

Tumour necrosis factor (TNF)-related apoptosis inducing ligand (TRAIL) is a promising pro-apoptotic protein that rapidly induces extrinsic apoptosis in tumor cells while sparing normal cells. As a homotrimeric form, TRAIL binds to its cognate death receptor 4 (DR4) or DR5 on the target cell surface, resulting in the recruitment of the adaptor protein FAS-associated protein with death domain (FADD), in turn, the assembling and activation of caspase-8 and -10 at the death-inducing signaling complex (DISC) [1]. Then the activation of caspase-8 and -10 leads to activation of the effector caspase-3, ultimately resulting in apoptosis [2]. Its soluble recombinant N-terminus truncated form, namely rTRAIL, has been extensively tested as a cancer therapeutic agent in vitro and in vivo [3,4]. However, as a monotherapy, the clinical benefits of rTRAIL were quite limited, possibly due to its poor bioavailability and common cancer cell resistance [5]. To overcome these shortcomings, strategies for efficient TRAIL delivery and circumvention of TRAIL resistance are necessary.

Extracellular vesicles (EVs) are submicron-sized membrane-enclosed vesicles released by numerous cell types, and are emerging as a novel delivery vehicle for therapeutic agents. They can be harvested from biofluids including blood, urine, saliva, amniotic fluid or supernatants of cell culture, exhibiting variable sizes, including small EVs of roughly 50–150 nm in diameter (also referred to as exosomes) and larger microvesicles of 30–1000 nm [6]. EVs are now under extensive evaluation as a therapy vector considering their unique advantages, such as the ability to infiltrate and penetrate tissue and blood-brain barrier, good stability in vitro and in vivo, modification flexibility, and good bioavailability with little toxicity and immunogenicity [6,7]. The safety of EV administration has been demonstrated by several phase I clinical studies with no grade II toxicity or maximal tolerated dose being found [8]. As an ideal drug delivery system, EVs can deliver not only soluble cargoes like microRNAs and mRNAs [9] but also membrane bound therapeutic molecules such as proteins and lipids [10]. 

It has been well demonstrated that TRAIL can be secreted via EVs by different types of cells that express TRAIL [10,11]. Importantly EV-carried TRAIL molecules (EV-T) may exist as membrane-bound oligomers, which are of higher efficiency for apoptosis induction. Thus, it is rationale to use EV-T instead of rTRAIL for cancer treatment to obtain better therapy efficacy.

Another strategy for improving efficacy can be combinational therapy of EV-T with other therapeutic agents, which can regulate the modulator expression levels in TRAIL signaling pathway and thus sensitizes TRAIL response in cancer cells [2]. It is now well known that some cancer lines are prone to DR-mediated apoptosis and are thus TRAIL sensitive. However other cancer cell types are not sensitive to DR stimulation and thus resistant to TRAIL. To exploit TRAIL for successful apoptosis induction in these resistant cells, the activation of mitochondrion-mediated apoptosis pathway is required as well [2]. Dinaciclib (SCH 727965) is such a promising chemotherapeutic drug under clinical trials showing regulatory activity on TRAIL pathway components and triggering mitochondrial pathway apoptosis in cancer cells [12,13]. This drug is a novel potent cyclin-dependent kinase (CDK) inhibitor that inhibits CDK1, CDK2, CDK5 and CDK9 activity in vitro at nanomolar concentrations [14]. Dinaciclib has been shown to trigger apoptosis and cell cycle arrest in over 100 tumor cell lines in vitro and induce regression of established solid tumors in a range of mouse models [14]. Clinically this drug has shown encouraging benefits for the treatment of relapsed/refractory chronic lymphocytic leukemia (CLL) patients [15].

In this study we sought to investigate whether TRAIL delivery by EVs is more efficient for apoptosis induction in cancer cells. And also, we tested the combinational therapy with EV-T and dinaciclib for TRAIL-resistant tumors in a murine xenograft model, aiming to define a safe and highly effective therapeutic window as a base for future clinical application.

## 2. Results

### 2.1. Generation and Characterisation of TRAIL-loaded EVs

**Figure 1:**
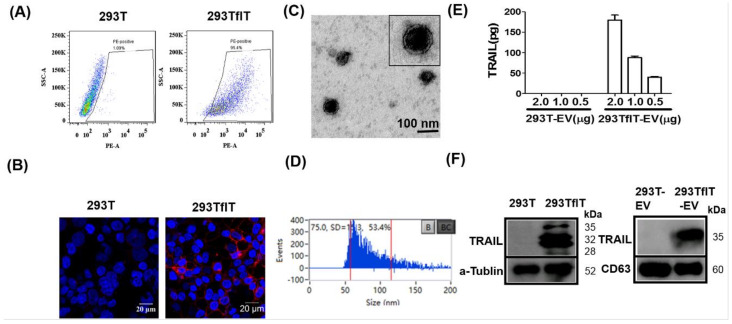
Generation and characterisation of TRAIL-loaded EVs (TRAIL-EVs). (**A**) Flow cytometry analysis of TRAIL expression in 293T cells transfected by empty virus (293T) or TRAIL-expressing lentiviruses (293TflT); (**B**) Immunofluorescent detection of TRAIL expression (red) by a PE-anti human TRAIL antibody in 293T and 293flT cells with nuclei labeled as blue by DAPI; (**C**) Electron microscopy of EVs isolated and purified by ultra-centrifugation of 0.22 µm filtering cleared supernatant of 293TflT cells grown in EV-depleted FBS containing medium; (**D**) Assessment of size distribution of isolated EVs by a Flow Nanoanalyzer that can detect nanoparticles with size ranging from 7 to 1000 nm; (**E**) Measurement of TRAIL expression levels in EVs derived from 293T (293T-EV) or 293TflT(293TflT-EV) using a commercial TRAIL-specific ELISA kit. Values are mean ± S.E.M (*n* = 3). (**F**) Western blotting detection of TRAIL, tetraspanin CD63 and loading control protein alpha-tubulin in EVs or cellular lysates; for each sample 10 µg of cellular or EV proteins were analyzed.

Previous studies have revealed that TRAIL could be secreted via EVs by cells that express TRAIL [16]. Thus, we could use TRAIL-overexpressing cells to produce TRAIL-loaded EVs (TRAIL-EVs). The 293T cell line was used to produce TRAIL-EVs in this study considering its plausibility for easy transduction and expansion and thus readily approachable mass production of EVs. Human full length TRAIL(flT)-expressing lentiviruses were prepared using the TRAIL lentiviral vector constructed previously [17] and used to transduce 293T cells at a multiplicity of infection (MOI) as 3. The flT transduced cells were designated 293TflT. Flow cytometry analysis showed that over 95% of 293TflT cells were stained TRAIL positive by a PE-conjugated anti-TRAIL antibody, whereas the control empty viruses transduced cells were negative (Figure 1A). As shown by immunofluorescent labeling in Figure 1B, TRAIL was successfully expressed in 293TflT cells.

Having created TRAIL-expressing 293TflT cells, we next examined TRAIL secretion via EVs by these cells. EVs were isolated from supernatant of cell culture by sequential centrifugation, 0.22 μm filtration and ultracentrifugation. The isolated EVs were examined with transmission electron microscopy (TEM), which revealed a membrane-enclosed vesicle structure of approximately 50–80 nm in diameter (Figure 1C). Also, EVs were analyzed for size distribution by the latest nano-flow cytometry and varying sized vesicles between 50–200 nm in diameter were observed (Figure 1D). However, majority of the EVs were among 50–80 nm range with an average vesicle size of 75 nm, which is consistent with the TEM observation. The expression of TRAIL in EVs was assessed by using a highly specific commercial ELISA. The obtained results showed that 95.2 ± 2.5 pg of TRAIL was carried by 1 μg of 293TflT-derived EVs; by contrast, control viruses transduced cell-derived EVs showed no detectable TRAIL expression (Figure 1E). Finally, TRAIL expression was further examined on cells and EVs by immunoblotting analysis (Figure 1F), and the whole immunoblotting results were shown as Figure S1. Three molecular forms of cellular TRAIL were detected in the 293TflT lysates that of ∼35 kDa and ∼32 kDa, and that of ∼24 kDa, corresponding to a cleaved form [18]. By contrast, TRAIL presented by 293TflT-EVs, namely EV-T, was resolved as a single band of ~35 kDa. Combination of ultracentrifugation with 0.22 μm filtration preferentially isolated small EVs (exosomes). The readily detection of tetraspanin CD63 (Figure 1F, right panel) suggests the isolated vesicles are mostly exosomes. However, microvesicles (MVs) of smaller sizes (below 220 nm) cannot be separated with exosomes by our isolation procedure. We thus name our preparation as EVs according to the Minimal Information for Studies of Extracellular Vesicles 2018 guideline (MISEV2018) [19].

**Figure 2:**
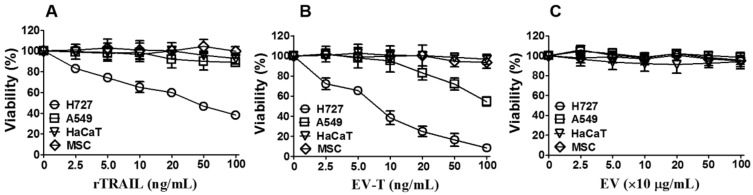
EV-T showed specific cytotoxicity to the highly TRAIL-resistant A549 line. Four cell lines (H727, A549, MSC and HaCaT) were treated with 2.5, 5, 10, 20, 50 and 100 ng/mL of rTRAIL (**A**), or EV-bound TRAIL (EV-T) (**B**), or EVs at indicated protein concentrations (**C**) for 24 h, respectively, followed by cell proliferation and viability assessment by Cell Counting Kit-8 (CCK-8). IC50 value for H727 and A549 lines treated by EV-T was determined as 8.1 ng/mL and 99.5 ng/mL by non-linear regression analysis, respectively. Values are mean ± S.E.M (*n* = 3).

To determine if EV-T is superior to rTRAIL for cancer cell killing, four cell lines (H727, A549, MSC and HaCaT) were chosen and tested for their responses to EV-T, rTRAIL and EV treatment, respectively. Both A549 and NCI-H727 (H727) are human non-small cell lung cancer (NSCLC) cell lines. Previous study showed that H727 is sensitive to TRAIL, whilst A549 is highly TRAIL resistant [17]. HaCaT, a spontaneously immortalized human keratinocyte line, and MSCs, primary human mesenchymal stem cells, were both used as control normal cells. The treated cells were assessed for their viability by Cell Counting Kit (CCK)-8. As expected, rTRAIL induced cell death in H727, but not in A549, HaCaT and MSC (Figure 2A). However, not only the sensitive H727 but also the resistant A549 cells were responsive to EV-T treatment in a dose-dependent manner, whilst normal MSC and HaCaT cells were not affected for viability (Figure 2B). Of note, EVs from parental 293T cells did not interfere with the viability of any tested cells (Figure 2C), indicating that it is TRAIL carried by EV but not EV itself to convey the cytotoxicity. Moreover, it has to be pointed out that EV-T only partially overcomes cancer resistance because its IC_50_ value for A549 appears to be 99.5 ng/mL, which is more than 10-fold higher than that in H727 cells (8.1 ng/mL) (Figure 2B). This suggests that although EV-T is effective to kill resistant cancer cells, further sensitization is needed for EV-T to obtain even better efficacy for cancer treatment.

### 2.2. Co-Treatment with EV-T and Dinaciclib Demonstrates Enhanced Cytotoxicity to Cancer Cell Lines that Express High Level of DR5

**Figure 3:**
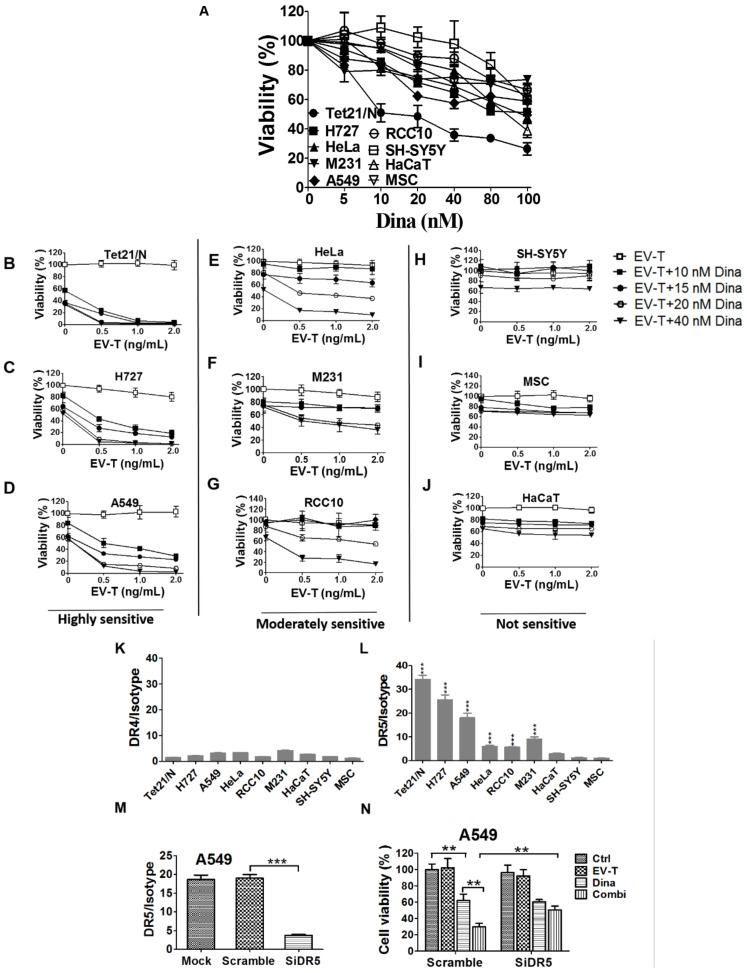
Combination of EV-T and dinaciclib shows enhanced cytotoxicity to cancer cell lines expressing DR5. (**A**) Evaluation of cell growth inhibition by dinaciclib on a panel of 9 cell lines. Cells were treated by dinaciclib at indicated concentrations for 24 h and then analyzed for cell viability and proliferation by CCK-8 kit. (**B**–**J**). Assessment of combinational treatment effects with low dose of EV-T and dinaciclib at indicated concentrations on cell lines tested in (**A**). The examined 9 lines were classified as three groups according to their sensitivity to the co-treatment: highly sensitive, moderately sensitive and not sensitive. (**K**,**L**). The detection of cellular surface expression of death receptor -4 (DR4) and -5 (DR5). The panel of 9 cell lines were labeled with PE-conjugated mouse mAbs against human DR4 or DR5 or with a mouse PE-IgG isotype, followed by flow cytometry analysis for the expression of DR4 (**K**) and DR5 (**L**), respectively. Expression levels were presented as the median fluorescence intensity (MFI) of DR4/DR5 labeling normalized by isotype staining. (**M**) Knockdown of DR5 expression in A549 cells. (**N**) Knockdown of DR5 rescued A549 cells from the enhanced killing of EV-T by dinaciclib combination. All values are mean ± S.E.M. (*n* = 3). ** *p* < 0.01, *** *p* < 0.001 versus MSC or as indicated, Student’s T test.

Having established that EV-T partially overcomes TRAIL-resistance of cancer cells, we next addressed the feasibility of using dinaciclib to boost its effect, since an increasing volume of data has demonstrated that combination therapy of dinaciclib with other drugs may promote therapeutic efficacy while reducing adverse side effects [20]. A panel of seven established cancer cell lines and two normal cell lines (MSC and HaCaT) were tested. The cancer panel includes five types of cancer consisting of two lung cancer lines, A549 and H727; two human neuroblastoma lines, SH-SY5Y and SH-EP Tet21/N-Myc (Tet21/N); one renal cancer line, RCC10; one human cervical cancer line, Hela; and one breast cancer line, MDAMB231 (M231). Firstly, these cell lines were tested for responses to dinaciclib alone treatment. As shown in Figure 3A, the inhibitor was cytotoxic to both cancerous and normal cell lines, although relatively higher sensitivity seen in cancer lines, indicating cautiously determined proper dosage must be essential for its future clinical application. 

Next, cells were co-treated with a range of low dose EV-T (0–2.0 ng/mL) and dinaciclib (10–40 nM), respectively, followed by cell viability assessment by CCK-8. The obtained results showed great response variances within the 9 lines (Figure 3B–J), which can be grouped accordingly into those that were highly sensitive to co-treatment (Figure 3B–D, group-1: Tet21/N, H727 and A549, cell death >85% by co-treatment with 1.0 ng/mL EV-T and 20 nM dinaciclib), those moderately sensitive (Figure 3E–G, group-2: Hela, M231and RCC-10, death ~40–60%), or those not sensitive (Figure 3H–J, group-3: SH-SY5Y, HaCaT and MSC, death ~10–25%). No dose-dependent response was examined in group-3 and the observed inhibition was obviously due to dinaciclib activity. In addition, EV-T and rTRAIL were compared for their efficacies to kill resistant A549 cells when in combination with dinaciclib and the obtained results showed that EV-T is more efficient than rTRAIL (Figure S2). These data showed that the combination of EV-T and dinaciclib is markedly cytotoxic to some cancer lines, whilst sparing normal cells like MSC and HaCaT and certain cancer lines like SH-SY5Y as well.

The DR4/DR5 binding by TRAIL is the initial step of TRAIL signaling pathway. Thus, the observed cytotoxicity by co-treatment with dinaciclib and EV-T may be associated with cellular surface DR4/DR5 expression levels. To validate such a likely correlation, the above 9 lines were examined for cell surface DR4 and DR5 expression levels by FACS. As shown in Figure 3K, all tested cell lines expressed similarly low levels of DR4 with median fluorescence signal intensity (MFI) ranging from 1.5 to 4.8 only. By contrast, the nine lines demonstrated greatly varied DR5 levels ranging from 1.5 to 34.2 (Figure 3L), and could be accordingly grouped into those of high DR5 levels (MFI 18.0-34.2, group -1 including Tet21/N, H727 and A549), those of moderate DR5 levels (MFI 5.5–9.3, group-2 including Hela, RCC-10 and M231), and those of low DR5 levels (MFI 1.5–3.1, group-3 including SH-SY5Y, HaCaT and MSC). Interestingly these three DR5 expression groups exactly matches aforementioned three co-treatment sensitivity groups, strongly suggesting a correlation between cellular surface DR5 levels and cell sensitivity to co-treatment by EV-T and dinaciclib. The possible effects of therapeutic agents on DR4/DR5 expression were also examined by FACS and the results (see Figure S3) showed neither DR4 nor DR5 levels was affected by EV-T or/and dinaciclib treatment in A549 cells. We further revealed that knockdown of DR5 expression in A549 cells by any one of 3 designed siRNAs (Table S1) against DR5 (Figure 3M shown for one siRNA only) can abrogate the enhanced killing effects of EV-T by dinaciclib (Figure 3N), which confirmed the necessity of DR5 expression for EV-T mediated cytotoxicity enhancement.

### 2.3. EV-T Enhances the Cell Cycle Arrest at G2/M Phase Induced by Dinaciclib in A549 Cells

Dinaciclib has recently been shown to induce cell cycle arrest in lymphoma Raji cells [13]. It is thus worth of testing if such effect is also available in the cancer lines used in this study. To this end, cell cycle distribution assay was performed for MSC, H727 and A549 cells. Cells were cultured and treated for 24 h by 1.0 ng/mL EV-T alone, 15 nM dinaciclib alone or the combination of the two agents, respectively, followed by PI staining and FACS analysis.

**Figure 4:**
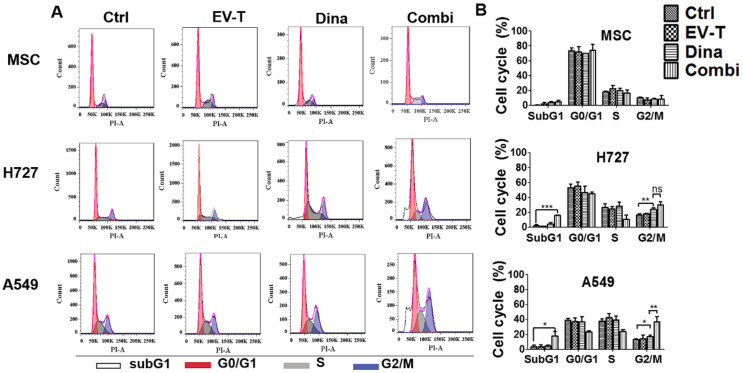
EV-T in combination with dinaciclib induced enhanced cell cycle arrest at G2/M phase in A549 cells. (**A**) Representative DNA content analysis of A549, H727 and MSC cells that were treated for 24 h by vehicle control (Ctrl), 1.0 ng/mL EV-T (EV-T), 15 nM dinaciclib (Dina), or the combination of EV-T and Dina (Combi), respectively. After treatments cells were stained with PI and analyzed using flow cytometry. Cells with 2n DNA are in G1-phase, 4n DNA are in G2/M-phase, and 2n-4n DNA are in S-phase. Cells that failed to be in cell cycle phases are considered dead (SubG1). (**B**) The cell cycle distribution values were obtained from three independent experiments and represented as mean ± S.E.M. The significance values were calculated by Bonferroni multiple comparison statistic test (* *p* < 0.05, ** *p* < 0.01, *** *p* < 0.001).

As shown in Figure 4, dinaciclib induced cell cycle arrest in G2/M phase in H727 and A549 lines but not in MSCs. Combined with EV-T treatment, the arrest effect was further enhanced in A549 but not in H727, indicating that the resistant A549 line may be selectively sensitive to the combination treatment. Consistent with cell viability assessment and apoptosis assay, the combination treatment also caused specific cancer cell death, as manifested by significant increase of SubG1 cells detected in A549 and H727 but not in MSC cells. Thus EV-T can be combined to enhance the effects of dinaciclib on cancer cell growth inhibition and killing.

### 2.4. EV-T in Combination with Dinaciclib Induced Strikingly Enhanced Apoptosis in Cancer Cells

One physiological role of TRAIL is to activate cellular surface DR4/DR5 and induce extrinsic apoptosis in target cells. Based on the above observed marked cytotoxicity of EV-T and dinaciclib co-treatment on cancer cells, we infer that the combinatorial treatment may stimulate enhanced apoptosis responses as well. To validate such an assumption, apoptosis assay was carried out for MSCs and A549 cells, which were treated by vehicle, 1 ng/mL of EV-T (corresponding to 10 µg/mL TRAIL-EVs) or/and 15 nM dinaciclib, anti-TRAIL antibody T3067 (Ab), or co-treatment with EV-T, dinaciclib and Ab (Combi+Ab), respectively.

**Figure 5:**
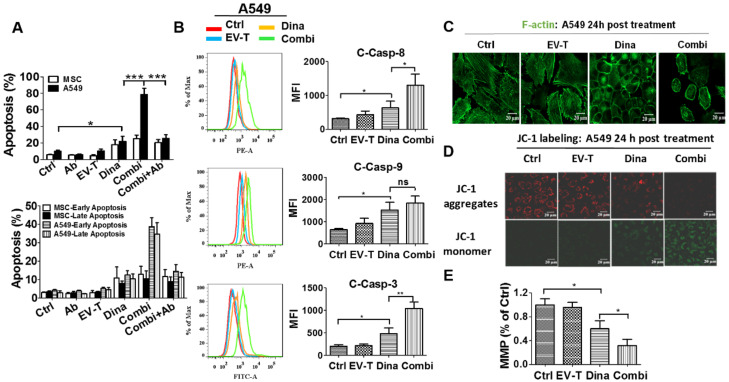
EV-T synergizes with dinaciclib to induce strikingly augmented apoptosis in A549 cells. (**A**) Apoptosis assessment by AF488-Annexin V/PI staining assay for A549 and MSC cells treated by saline (Ctrl), 100 ng/mL TRAIL-neutralizing monoclonal antibody (Ab) (T3067, Sigma-Aldrich), EV-T at 1.0 ng/mL (EV-T) or/and dinaciclib at 15 nM (Dina) (Combi), and Combi plus Ab (Combi+Ab) for 24 h, respectively; (**B**) Comparison of expression levels of activated caspase-8 (C-Casp-8), -9 (C-Casp-9), and -3 (C-Casp-3) by their related PE-, or FITC-conjugated inhibitor labelling combined with flow cytometry analysis in A549 cells that were treated like in A. (**C**) Confocal fluorescent microscopic observation of actin cytoskeleton in A549 cells treated like in A, followed by FITC-phalloidin staining to show F-actin (green). The disruption of actin cytoskeleton in either dinaciclib or combination treatment cells indicates apoptosis occurrence [21]. (**D**). Detection of mitochondrial membrane potential (MMP or Δψm) change in A549 cells. Cells were treated like in A for 24 h, followed by JC-1 probe labeling and confocal fluorescent microscopic imaging. The loss of Δψm was indicated by the decrease of JC-1 aggregate abundance (red fluorescence) and the consequent increase of JC-1 monomer abundance (green fluorescence). (**E**) The fluorescence intensity ratio of JC-1 aggregates to monomers was calculated as an indicator of the mitochondrial membrane potential (MMP). MMP is shown relative to control (Ctrl), the value for which was set to 1. All values are mean ± S.E.M (*n* = 3). ns, not significant, * *p* < 0.05, ** *p* < 0.01, *** *p* < 0.001 versus Ctrl or Dina, analyzed by Students’ T test.

The obtained results (Figure 5A) demonstrated that low dose of dinaciclib alone but not low dose of EV-T alone induced detectable marginal apoptosis in both MSC and A549 cells. Interestingly the combination treatment stimulated markedly enhanced apoptosis induction in A549 cells but not in MSCs in comparison with dinaciclib alone treatment. In addition, the enhanced apoptosis induction by co-treatment could be abrogated by the addition of TRAIL-neutralization antibody (Figure 5A), indicating the extrinsic apoptosis pathway activation via EV-T.

Apoptosis activation was confirmed by the detection of significantly increased activation of caspase-8, -9 and -3 by dinaciclib and the further enhancement of activation for caspase-8 and -3 by combinational treatment with EV-T in A549 cells (Figure 5B). Moreover, the disruption of actin cytoskeleton as well as loss of mitochondrial membrane potential were also observed in A549 cells (Figure 5C–E) but not in MSCs that were treated by either dinaciclib or its combination with EV-T, which further confirmed the apoptosis-inducing activity of dinaciclib as well as the drastic augmentation of its efficacy by EV-T in certain cancer cells. The decrease of MMP indicates the initiation of caspase-9 activation but will not necessarily results in enhancement of the cleavage of caspase-9 since this process is further subject to downstreatm regulation by other factors such as XIAP, CIAP and APAF. Collectively EV-T and dinaciclib appear to act synergistically, but this relationship needs further experiments to confirm.

### 2.5. Combinational Treatment by Dinaciclib and EV-T Regulates the Expression of TRAIL Apoptosis Pathway Modulators in A549 Cells

**Figure 6:**
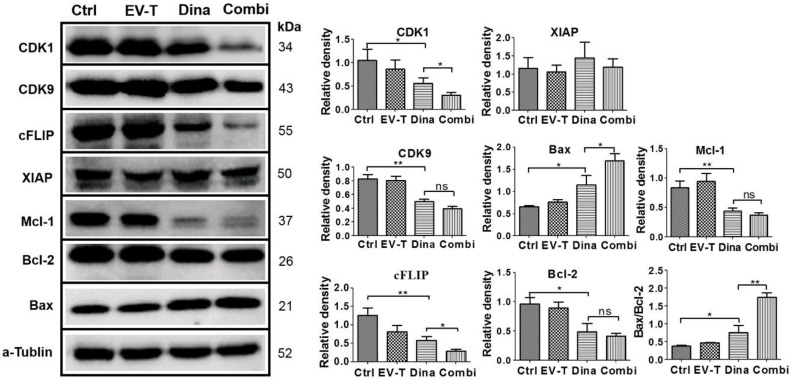
Expression detection of TRAIL signaling pathway modulators in A549 cells by immunoblotting. Cells were treated by vehicle (Ctrl), 1.0 ng/mL of EV-T (EV-T) or/and 15 nM of dinaciclib (Dina) (Combi) for 24 h, respectively, followed by Western blotting analysis with antibodies against CDK1, CDK9, cFLIP, XIAP, Mcl-1, Bcl-2, Bax and a–tubulin. The relative mean densitometry values of examined proteins were determined from three independent experiments and were shown as mean ± S.E.M (*n* = 3). The ratio of Bax/Bcl-2 expression levels was also shown, and its significant increase indicates mitochondrial pathway apoptosis was triggered. ns, not significant, * *p* < 0.05, ** *p* < 0.01, Students’ T test.

It is now known that TRAIL signaling pathway is regulated by intracellular pro- and anti-apoptotic proteins [2]. Theoretically, expression changes of TRAIL pathway modulators could affect TRAIL sensitivity in target cells. Having revealed that the combination of dinaciclib with EV-T stimulates drastically enhanced apoptosis in some cancer lines like A549, we therefore, next investigated whether already known anti-apoptotic proteins cFLIP, XIAP, Bcl-2, CDK1and CDK9, and pro-apoptotic protein Bax, are regulated by EV-T, dinaciclib or combination of the two agents in A549 cells. The treated cells were analyzed for target protein expression by immunoblotting and obtained results were shown in Figure 6.

The complete immunoblotting results are shown in Figure S4. While EV-T at 1 ng/mL failed to show any significant influence and XIAP was not affected by any treatments, CDK1, CDK9, cFLIP, Mcl-1 and Bcl-2 protein levels were all downregulated by dinaciclib treatment at 15 nM, and the downregulation was further boosted for CDK1 and cFLIP but not for CDK9, Bcl-2 and Mcl-1 by the addition of EV-T to dinaciclib treatment. By contrast, Bax protein level was upregulated by dinaciclib treatment and the upregulation could be further enhanced by the combination of EV-T. The Bax/Bcl-2 ratio was also shown to be increased by dinaciclib and further upregulated by co-treatment, indicating enhancement of apoptosis induction. These data suggest that combination therapy with dinaciclib and EV-T could reprogram cells to change TRAIL apoptosis pathway modulator expression profiles, shifting A549 behavior from TRAIL-resistant to TRAIL-sensitive.

### 2.6. Combination Therapy with EV-T and Dinaciclib Eradicated the Subcutaneous Growth of Resistant A549 Tumors in a Murine Xenograft Model

Cancer resistance is one of the main obstacles with respect to the clinical application of TRAIL. In vivo experiments were carried out to test the combination efficacy of dinaciclib with EV-T or rTRAIL for overcoming the resistance of A549 tumor.

**Figure 7:**
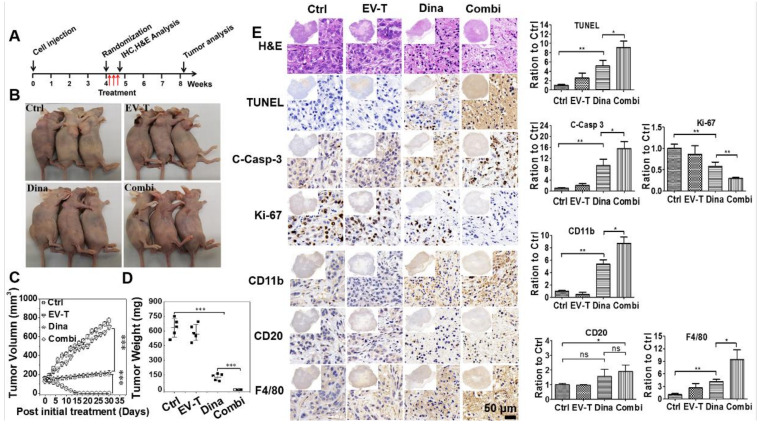
EV-T and dinaciclib combination therapy eradicates established subcutaneous lung tumors in vivo. (**A**) Experimental treatment schedule is shown. (**B**) Representative images of experimental mice at day 10 post last treatment. Each treatment comprises of intratumoral injection per animal of saline vehicle (Ctrl), 4.5 ng EV-T (corresponding to 50 μg EVs) (EV-T), 160 μg dinaciclib (Dina), or combination of 4.5 ng EV-T and 160 μg dinaciclib (Combi), total three injections performed with a 48 h interval for each animal. (**C**) Tumor growth/volume curves after various treatment. (**D**) Statistical comparison of tumor weight for various therapies at the end of the experiment. (**E**) H&E and immunohistochemistry (IHC) analyses of subcutaneous A549 tumors harvested 12 h post the final treatment. Cell proliferation marker Ki-67, apoptosis (activated caspase 3 short for C-Casp-3, TUNEL) as well as tumor infiltrating of murine immune cells including natural killer (NK) (biomarker CD11b), B lymphocytes (CD20) and macrophages (F4/80) were examined. Quantification histogram of IHC positive staining signal intensity for each marker molecule is shown with saline control value set as 1. Ten different but histologically similar fields were selected for quantification for each sample and the slides were analyzed quantitatively using Image-pro plus 6.0 software. All values are mean ± S.E.M (*n* = 3), ns, not significant, * *p* < 0.05, ** *p* < 0.01, *** *p* < 0.001, Students’ T test.

As indicated in Figure 7A, five million A549 cells were subcutaneously injected into each Balb/c nude mouse for tumor growth. About 4 weeks post A549 injection, when tumor nodule growth reached around 100 mm^3^, treatment by intra-tumor injection of therapeutic agents EV-T, dinaciclib, combination of EV-T and dinaciclib, combination of rTRAIL and dinaciclib, or vehicle was started. The EV-T dose is 4.5 ng TRAIL per injection (0.25 µg/kg), corresponding to ~50 µg EVs, which is 3-fold lower than the reported maximal tolerated EV dose (200 µg) and much lesser than the previously observed *in vivo* effective rTRAIL dosage (100 µg) in murine models in which i.p. injection was used [22]; the dinaciclib dose was 160 µg per injection (8 mg/kg) that is much lower than previously observed in vivo effective dose (50 mg/kg) in a murine model of breast cancer with i.p. injection of the drug by others [23], and total three injections were carried out for each mouse with a 48 h interval. The combination therapy consists of dinaciclib at 8 mg/kg and EV-T or rTRAIL at 0.25 µg/kg. Treatment effects were assessed by measuring tumor volume changes every 48 h and weighing resected tumors at the experimental endpoint. Comparing with the control saline injection, low dose of EV-T administration only led to a marginal inhibition effect on tumor growth (maximal TVI 15%). By contrast, either dinaciclib monotherapy or its combination with EV-T or with rTRAIL induced rapid and persisting inhibition of tumor growth, causing ultimate TVI 72 %, 100 % (Figure 7B,C) and 79 % (Figure S5), respectively. Of note, the combination therapy by EV-T and dinaciclib obtained complete response, showing initial tumor shrinkage 2 days after the last treatment and full tumor regression around day 14. The combination of low dose of EV-T and dinaciclib is significantly more efficient than that of rTRAIL and dinaciclib, which only showed slightly promoted inhibition of tumor growth than dinaciclib alone treatment, suggesting EV-T is superior to rTRAIL when combined with dinaciclib for tumor therapy. Accompanied with the tumor shrinkage and regression, the EV-T combination therapy group showed slight decline of body weight between treatment day 4 to day 8, then gradually grew back close to initial levels (Figure S6). The body weight changes may reflect the rapid tumor regression as well as the possible slight growth inhibition by dinaciclib. Weighing of resected tumors at the endpoint confirmed the significant inhibition of tumor growth by dinaciclib and the tumor eradication by the combinational treatment (Figure 7D).

Lesions removed 24 h post the last treatment were analyzed by H&E staining and immunohistochemistry (IHC). H&E staining revealed the accumulation of pyknotic nuclei in tumors treated by dinaciclib or combination therapy, indicating apoptosis occurred within these tumors. Subsequently apoptosis induction was confirmed by tunnel staining and the detection of pro-caspase-3 activation (Figure 7E). Tunnel staining revealed that dinaciclib induced significant apoptosis in tumors in comparison with vehicle treatment, and this effect was further strikingly promoted by combination with EV-T. Examination of activated caspase-3 expression confirmed these results. Moreover, detection of the cell proliferation marker Ki67 revealed that dinaciclib alone treatment caused significant tumor growth attenuation and its combination with EV-T caused further enhanced inhibition. To evaluate the therapy safety, animal organs including heart, liver, spleen, lung, kidney and intestine were removed at the experimental endpoint and examined by H&E staining for the control and combination treatment groups, respectively. No gross abnormalities or other signs of toxicity such as necrosis, apoptosis and fibrosis were observed (Figure S7).

Nude mice are athymic but not immunologically neutral, and can display natural killer (NK) and macrophage cell reactivity as well as produce immunoglobulins by B cells [24]. Some reports demonstrated the effects of dinaciclib and TRAIL on immune cells via inducing cell infiltration into tumors [25,26,27]. Therefore, it will be interesting to examine if residual nude mice immune cells were involved in the killing and clearance of A549 xenograft tumors by EV-T and/or dinaciclib treatment. To this end, consecutive sections of tumor lesions that were resected 24 h post last treatment were stained by antibodies against cell surface markers for mature murine NK cells (CD11b) [28], B cells (CD20) [29] and macrophages (F4-80) [30], respectively. As shown in Figure 7E, dinaciclib alone treatment stimulated significant infiltration of NK cells and macrophages in tumors, and the combination of EV-T further promoted the infiltration. Also, significant B cell recruitment and infiltration in tumors was induced by the combination therapy but not by dinaciclib alone treatment. While the underlying mechanism(s) for immune cell infiltration remain to be elucidated, the recruitment of active NK cells, B cells and macrophages to tumors may facilitate the killing and elimination of tumor lesions.

Collectively, these data demonstrated that dinaciclib is potent for sensitizing in vivo TRAIL response in cancers and its combination with EV-T can trigger strikingly enhanced apoptosis induction resulting in complete eradication of subcutaneous TRAIL-resistant A549 tumor growth.

## 3. Discussion

In this work we demonstrated: (1) that TRAIL-expressing 293T cells release TRAIL carried by EVs (EV-T), (2) that EV-T is highly efficient for selective apoptosis induction in cancer cells, (3) that CDK inhibition by dinaciclib drastically sensitizes TRAIL response in cancer cells, (4) that combination therapy with EV-T and dinaciclib led to complete eradication of subcutaneous A549 xenograft tumor growth without any adverse events observed.

### 3.1. Membranous TRAIL Carried by EVs Is Highly Efficient for Cancer Cell Killing

Extracellular vesicles (EVs) including exosomes and microvesicles are emerging innovative nano-sized drug delivery system (DDS) for cancer therapy [31]. EVs can be an ideal delivery vehicle for cancer therapeutic agents thanks to their many unique merits such as biological barrier penetration ability, tumor homing and organotropism, good bioavailability [10], innate stability and multi-drug loading capacity, little toxicity and immunogenicity [32]. Increasing evidences have demonstrated the great potential of EVs for delivery of therapeutic proteins and siRNAs for cancer treatment. For example, the fusion protein of cytosine deaminase (CD) and uracil phosphoribosyltransferase (UPRT) has been successfully expressed on transfected 293T cell-derived microvesicles, which synergized with chemo prodrug 5-fluorocytosine to induce nerve sheath tumor apoptosis and regression [33]. In a study with murine models, oncogenic Kras-targeted siRNA was loaded into mesenchymal stem cell-derived exosomes, which were then tested to confirm suppression of Kras in vitro and in vivo and a consequent increase in the survival of animals with pancreatic cancers [34].

Previously we have revealed that full length TRAIL was not only expressed on cell plasma membrane as membrane TRAIL but also secreted into supernatant of cell culture by TRAIL transfected MSCs, and importantly the membrane TRAIL is superior to soluble type (rTRAIL) for inducing apoptosis in cancer cells [17]. In this study we also observed that TRAIL can be secreted via EVs by TRAIL-overexpressing 293T cells and the EV carried membrane TRAIL (EV-T) can kill cancer cells with higher efficiency than rTRAIL. Actually TRAIL delivery as a membranous form on EVs is a natural approach for cells to direct the ligand to modulate the fate of target cells with high efficiency [35]. The high efficacy of membrane TRAIL may attribute to its potential to form oligomers or clusters due to the fluidic nature of phosphor-lipid bilayer membrane [35,36].

### 3.2. Combination Therapy with EV-T and Dinaciclib Is a Novel Therapeutic Strategy for Cancer Treatment

Now CDKs are well known to regulate cell cycle and apoptosis processes, and are often hyperactive in cancers owing to genetic or epigenetic events [37]. Therefore, CDKs have been extensively examined as good molecular targets for cancer therapy development from a long time ago. However broad-spectrum CDK inhibitors like flavopiridol were proven to be poorly selective to cancer versus normal cells. This is obviously due to the importance of CDKs in normal cell growth. To overcome the shortcomings, the second generation of CDK inhibitors like dinaciclib were developed, which are more selective and/or more potent. Dinaciclib has been under clinical trials as monotherapy and demonstrated encouraging but modest clinical activity on acute leukemias and myeloma showing a narrow therapeutic window possibly due to the lack of proper patient selection [38,39]. It is thus essential to find good predictive biomarkers and/or conduct combination therapy with other drugs to improve dinaciclib sensitivity for having a wider therapeutic window. However, it is very challenging to find positive predictors of response considering the complexity of CDK-cyclin pathway. It could be simpler to look for negative predictors. Cellular surface DR4/DR5 expression appears to be such a good negative predictor for the combination therapy of EV-T and dinaciclib. We observed that the higher DR5 expression is, the more sensitive cancer lines are to EV-T and dinaciclib co-treatment. Without good expression of DR4/DR5 as seen in normal MSC and HaCaT cells and in cancer line SH-SY5Y, cells were not sensitized to EV-T treatment by dinaciclib. Thus, dinaciclib is not expected to synergize with EV-T to work against tumors that express little DR4/DR5.

As monotherapy CDK inhibitors have not often led to complete tumor regression, especially in solid tumors. This may attribute to their preferential cytostatic activity rather than cytotoxic effect as seen previously [40]. Dinaciclib treatment often causes cancer cell arrest at G2/M phase as evidenced in this study and previously by others [13,23], a situation when cells are prone to apoptotic death, indicating other apoptosis-inducing drugs can be combined to obtain better results. For example, in glioma lines, dinaciclib alone inhibited proliferation only, however when combined with Bcl-xL silencing, it induced significant mitochondrial pathway apoptosis [12]. The combination of dinaciclib with cisplatin synergistically promoted cell cycle arrest and apoptosis in ovarian cancer [41]. Also, in this study, dinaciclib alone stimulated modest apoptosis only in A549 cells, but its combination with EV-T induced strikingly enhanced apoptosis, and importantly, led to complete A549 tumor regression in the nude mice xenograft model.

Dinaciclib blocks CDK1 that is needed for proliferation of normal cells. Therefore, the drug dose have many of the expected toxicities. In this study dinaciclib was shown to slightly affect viability of normal MSC and HaCaT cells in a dose-dependent manner, indicating low dosage is preferred for its clinical application. Fortunately, dinaciclib is generally less toxic than traditional chemo drugs. It exerts temporary and reversible growth inhibition on normal cells. In vitro experiments, normal cells arrested by dinaciclib resumed proliferating when the drug is removed [37]. In a phase I trial, dinaciclib in combination with rituximab was well tolerated in CLL patients and no dose-limiting toxicities were observed, although hematological, digestive and metabolic adverse events (AEs) were commonly observed [15]. In our study mice were treated by low doses of dinaciclib and EV-T and no AEs were observed, which demonstrated the unique advantage of the combination treatment. Our current observation together with findings from others thus strongly suggest that combination therapy is a good strategy for dinaciclib to improve its clinical efficacy and safety.

### 3.3. DR4 and/or DR5 Expression and Function Are Necessary for TRAIL-based Cancer Therapy

Apoptosis induction by TRAIL is generally ncancer specific possibly due to the fact that lower expression of DR4 and DR5 was detected in normal cells than in cancer cells [42]. However, a consistent correlation has never been found between the expression of DR4/DR5 and TRAIL sensitivity in cancer cell lines and tumors [43,44,45]. Our results in this study also demonstrated that TRAIL sensitivity is not necessarily associated with DR4 and DR5 expression levels. A549 cells expresses high level of DR5 but are highly resistant to TRAIL whilst H727 cells that express high level of DR5 do appear highly sensitive to TRAIL. However, A549 and other cancer cell lines with good DR5 expression can be drastically sensitized to TRAIL treatment by dinaciclib and the sensitizing extent seems to be proportional to DR5 levels. Thus, the good DR5 expression, possibly when DR4 expression is low, appears at least necessary for TRAIL sensitization by dinaciclib. This suggests that good DR5 and/or DR4 expression must be a criterion for patient selection for the EV-T and dinaciclib combination therapy in the future. The low expression of DR4 and DR5 seen in normal MSC and HaCaT not only explains their very low sensitivity to the combination treatment but also guarantees the good safety of the therapy. In addition, the low DR4/DR5 expression in SH-SY5Y line that is totally resistant to co-treatment of EV-T and dinaciclib further supports good DR5 and/or DR4 expression as a prequisite for TRAIL and dinaciclib combination- based cancer therapeutic regimes. The observation that knockdown of DR5 expression restored A549 resistance to co-treatment further proved the importance of DR expression for EV-T based therapy. Moreover DR5 and/or DR4 must be functional as well since inactivation of the two receptors by deletions or point mutations has been demonstrated to be closely associated with TRAIL resistance in cancers [46,47,48].

### 3.4. Mechanisms Associated with the Synergism between EV-T and Dinaciclib

**Figure 8:**
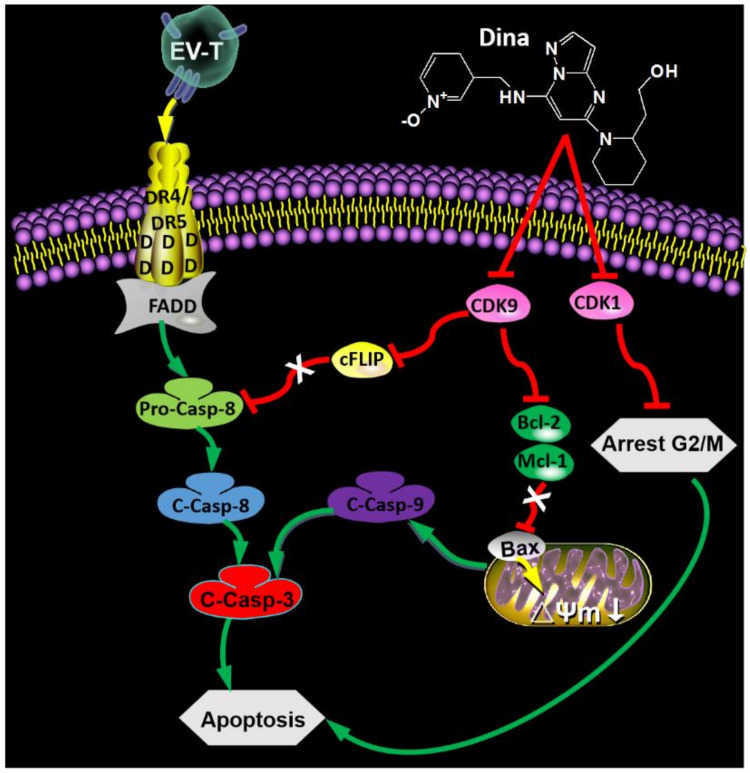
Schematic overview of synergism between EV-T and dinaciclib for cancer therapy. The EV-T ligand efficiently binds to functional receptors DR4 and DR5, inducing trimerization of the receptors. The cytoplasmic part of the DR4 and DR5 receptors contain death domain that enable recruitment of Fas-associated protein with death domain (FADD) and pro-caspase 8 (Pro-Casp-8), enabling cleavage and activation of pro-Casp-8 to its active form caspase 8 (C-Casp-8). Casp 8 activates downstream effector caspases such as caspase 3 (C-Casp-3). Dinaciclib (Dina) inhibits activity of CDK9 and CDK1 and downregulates their expression simultaneously. The CDK9 suppression results in downregulation of cFLIP and subsequent augmentation of Pro-Casp-8 activation by EV-T, decrease of Bcl-2 and Mcl-1 expression as well as upregulation of Bax, causing consequent decrease of mitochondrial membrane potential (Δψm) and activation of pro-caspase 9 (C-Casp-9), which takes part in activation of Casp-3. Activated effector caspases induce apoptosis. The suppression of CDK1 induces cell cycle arrest at G2/M phase in which cells are prone to apoptosis induction.

Dinaciclib caused inhibition of cell proliferation and tumor growth, possibly attributable to its suppression effects on both CDK1 activity and expression as observed in A549 cells in this study and previously by others as well [13,23,41]. Interestingly EV-T combination with dinaciclib further enhanced the downregulation of CDK1 by dinaciclib, for which the related mechanism is yet to be elucidated. Moreover as evidenced previously in ovarian cancer lines [41] and triple negative breast cancer [23], as well as in this study, CDK9 expression can be downregulated by dinaciclib, which may explain the observed augmentation of EV-T responses by the inhibitor. As a key component of the positive transcription elongation factor b (P-TEFb) complex [49], CDK9 is involved in apoptosis signaling regulation through modulating transcriptional expression of those anti- and pro-apoptotic factors [22]. As expected, the suppression of CDK9 activity and expression by dinaciclib caused decreases of anti-apoptotic proteins c-FLIP, Mcl-1 and Bcl-2 expression and increase of pro-apoptotic protein Bax expression in this study, subsequently resulting in strikingly promoted apoptosis induction by EV-T in cancer cells. It remains not clear why EV-T combination further downregulated c-FLIP expression whilst further increasing Bax expression in A549 cells. Taken together the underlying mechanisms for the observed markedly promoted growth inhibition and apoptosis induction by combined therapy can be the concomitant suppression of CDK1, CDK9, c-FIP, Mcl-1 and Bcl-2 and upregulation of Bax in cancer cells (Figure 8).

It is widely accepted that various immune cells participate in tumor behavior and fate determination [13]. We observed obvious tumor infiltration of abundant natural killer (NK) cells and macrophages and relatively fewer B cells following dinaciclib monotherapy or its combined therapy with EV-T in this study. Dinaciclib was previously shown to increase T cell infiltration and DC activation within mice tumors by eliciting immunogenic cell death (ICD) when combined with anti-PD1 therapy [26]. TRAIL may stimulate immune cell infiltration within tumors as well. ONC201, a TRAIL-inducing antitumor agent, was previously found to promote intratumoral NK cell recruitment [27]. Also the combined delivery of TRAIL and interleukin-12 (IL-12) by oncolytic adenoviruses resulted in infiltration of NK and antigen presenting cells within hepatocellular carcinoma [25].Therefore, it is not surprised that the combinatorial treatment of dinaciclib and EV-T induced remarkable tumor infiltration of immune cells in this study. However, it remains uncertain what roles these infiltrated immune cells play. Mature NK cells circulate in peripheral blood or reside in liver and spleen and are inherently cytotoxic to cancer cells [50]. Increasing evidences have shown anti-tumor effects of NK cells in vitro and in vivo [51]. Macrophages are differentiated monocytes that take part in the rapid and efficient clearance of apoptotic cells [52]. Generally, it is thought that there is little T cell-dependent activation events for B cells in nude mice. However growing evidence shows that B cells could be activated by non-canonical helper signals from macrophages, dendritic cells and other cells of the innate immune system [53,54]. Based on these published findings and also the fact that the combination therapy stimulated more tumor infiltrating of immune cells than dinaciclib monotherapy and led to the elimination of tumor growth, one can speculate that these infiltrated cells function to promote the killing and clearance of tumors cells in this study. Certainly, this hypothesis needs further verification.

## 4. Methods and Materials

### 4.1. Cell Culture

Cell culture reagents were purchased from Gibco (Life Technologies, Gaithersburg, MD, USA) unless otherwise stated. Seven cancer cell lines and three normal cell lines were used, including two human lung cancer lines, NCI-H727 and A549; one human breast adenocarcinoma line, MDAMB231 (M231); one human renal cancer line, RCC10; two human neuroblastoma cell lines, SH-SY5Y and Tet21/N; one human cervical cell line, Hela; three normal cell lines, umbilical cord derived mesenchymal stem cells (MSCs), keratinocyte of human skin (HaCaT) and HEK 293T line. M231, NCI-H727 and RCC10 were obtained from Cancer Research United Kingdom (London, UK). MSCs are a kind gift from Dr. Huang QB (PreGene Biotechnology Company, Ltd., Shenzhen, China). Other cell lines were purchased from Fuheng Biology (Shanghai, China). A549 and NCI-H727 were cultured in RIPM medium supplemented with 10% fetal bovine serum (FBS); 293T, SH-SY5Y and Tet21/N were cultured in the low sugar (1.0 g/mL) DMEM medium supplemented with 10 % FBS; HeLa, RCC10, M231 and HaCaT cells were cultured in the higher sugar (4.5 g/mL) DMEM medium with 10 % FBS; MSC was cultured in the DMEM/F-12 medium with 10 % FBS. All referred cell lines were cultured in a humidified atmosphere containing 5% CO_2_ at 37 °C.

### 4.2. Isolation of EVs

To prepare cell-derived EVs, 1.0 × 10^7^ 293T and TRAIL-expressing 293T (293TflT) cells were first cultured in DMEM medium containing 10 % FBS until cells reached 70–80% confluence; then the medium was changed to DMEM supplemented with 10 % EV-depleted FBS for a further 72 h. EV depletion was done by ultra-centrifugation of cleared FBS for 15 h at 120,000 *g* at 4 °C, using Optima L-80 XP ultra-centrifuge machine (Beckman Coulter, Brea, CA, USA) with rotor Ti-70, followed by supernatant collection. Culture supernatant conditioned by cells was collected and first sequentially centrifuged at 300 *g* and 2000 *g* each for 10 min at 4 °C to remove cells and debris, followed by vacuum filtering the medium through 0.22 μm filters (Merck Millipore, Darmstadt, Germany) to remove large vesicles, and finally ultracentrifuged for 2 h at 120,000 × *g* at 4 °C. The obtained EV products were washed twice with 0.22 μm membrane-filtered phosphate-buffered saline (PBS) and finally resuspended in PBS for storage in −80 °C until use.

### 4.3. Transmission Electron Microscopy 

Isolated EVs suspended in PBS were absorbed on formvar/carbon-coated nickel grids for 10 min and unbound EVs were washed away with PBS. Grids were then fixed in 4% poly-formaldehyde for 10 min and negatively stained with 0.3% uranyl acetate in 1.9% ethyl cellulose. Excess fluid was removed and grids were air dried before examination and imaging with a Tecnai T12 electron microscope (FEI, Eindhoven, The Netherlands). 

### 4.4. Size Distribution of EVs

EV size distribution was determined using the recently developed Nano-Flow Analyzer (nanoFCM, Xiamen, China) that allows the direct single-particle measurement of nano-sized entities including EVs for size and concentration [55]. Briefly, EVs (1 μg) were resuspended in 1 mL of filtered PBS at pH 7.4. Then two single-photon counting avalanche photodiodes (APDs) were used for the simultaneous detection of the side scatter (SSC) (bandpass filter: FF01 − 524/24) and orange fluorescence (bandpass filter: FF01 − 579/34) of individual EVs, respectively. Unless stated otherwise, each distribution histogram or dot-plot was derived from data collected for 1 min.

### 4.5. Immunofluorescence

Localization of TRAIL in cells was examined by means of immunofluorescence staining. Cells were grown on chamber slides for 2 days, fixed with 4% paraformaldehyde, permeabilized in 0.1% tween-20-containing buffer, blocked in PBS containing 10% FBS and 0.1% tween-20 and then stained with the PE-conjugated mouse anti-human TRAIL monoclonal antibody (Abcam, Cambridge, UK). The FITC conjugated phalloidin (Solarbio, Beijing, China) was used for labeling filamentous actin (F-actin). The nuclei of stained cells were located with DAPI (Solarbio) and then viewed and imaged by confocal microscopy (LSM800, Zeiss, Jena, Germany).

### 4.6. Cell Proliferation and Viability Assay

Assessment of cell proliferation and viability was performed using the Cell Counting Kit 8 (CCK-8) (Dojindo, Kumamoto, Japan). Briefly, 1 × 10^4^ cells were seeded in 96-well tissue culture plates in cell culture medium containing 10% FCS 12 h before treatment with EV, rTRAIL, EV-T or/and dinaciclib for 24 h, respectively, followed by CCK-8 analysis following the manufacturer’s instructions. Assays were performed in triplicate. Spectrophotometric absorbance at 450 nm was measured using a microplate reader (model 2300, PerkinElmer, Boston, MA, USA), and the percentage of surviving cells is calculated according to the equation: viability of cells (%) = (A*_sample_*-A*_blank_*)/(A*_control_*-A*_blank_*) × 100. A*_sample_*, A*_blank_* and A*_control_* represent UV absorption at 450 nm for cells treated by therapeutic agents, culture medium, or vehicle, respectively. Data were presented as mean ± S.E.M (*n* = 3). Dose-response curves were generated from triplicate, six-point serial dilutions of test agents and related IC_50_ values were derived by non-linear regression analysis. Synergism is defined as the interaction of discrete agents such that the total effect is greater than the sum of the individual effects in this study.

### 4.7. Cell Cycle Distribution Analysis

Cells were cultured and treated with vehicle, 1.0 ng/mL EV-T or/and 15 nM dinaciclib for 24 h. Then cells were harvested and washed twice with cold PBS, then fixed with ice-cold 70% ethanol for overnight at 4 °C. After centrifugation at 2000 *g* for 10 min, cells were washed twice with PBS and resuspended in 0.5 mL staining buffer containing 50 μg/mL PI and 100 μg/mL RNase A, incubated at 37 °C for 0.5 h. Cells were spun down by centrifugation at 2000 *g* for 10 min, washed twice with PBS and resuspended with 0.5 mL staining buffer without PI and RNase A and detected by FCM. Fluorescence was measured at an excitation wavelength of 480 nm through a FL-2 filter (585 nm). Data were analyzed using FlowJo 7.6 software (Becton Dickinson, Ashland, OR, USA).

### 4.8. Apoptosis Assay

For apoptosis assay, A549 and MSC cells were seeded with a density of 0.2 × 10^6^ cells/well and 0.1 × 10^6^ cells/well in 12-well plates, respectively. After settling down for overnight, cells were treated by vehicle or therapeutic agents for 24 h. Then both floating and adherent cells were stained with FITC-conjugated Annexin V and propidium iodide (Bestbio, Shanghai, China) and were assessed by means of flow cytometry (FACS Calibur; Becton Dickinson). Besides EV-T and dinaciclib, the TRAIL-neutralizing antibody T3067 (Ab) was also tested on MSC and A549 cells, either alone or together with co-treatment by EV-T and dinaciclib (Combi+Ab) to prove extrinsic apoptosis induction by EV-T. Annexin V^+^/PI^-^ and Annexin V^+^/PI^+^ cells were considered to have undergone early and late apoptosis, respectively, and Annexin V^−^/PI^−^ cells were regarded as live cells. 

### 4.9. Detection of Cleaved Caspases-8, -9 and -3

To detect expression of activated caspases-8, -9 and -3, A549 cells were cultured and treated with vehicle, 1.0 ng/mL EV-T or/and 15 nM dinaciclib for 24 h, respectively. Cells were then harvested and stained with the active caspase-8 inhibitor Red-IETD-FMK (K198-25, Bio-Vision, Zurich, Switzerland), active caspase-9 inhibitor Red-LEHD-FMK (K199-25, Bio-Vision) or active caspase-3 inhibitor FITC-DEVD-FMK (K183-25, Bio-Vision), respectively, according to the manufacturer’s instructions and analyzed by means of flow cytometry.

### 4.10. Knockdown of DR5 Expression by siRNA

Three duplex siRNAs against human DR5 gene (SiDR5) and one control siRNA (Scramble, Guangzhou, China) were designed and synthesized by IGE Biotechnology (Guangzhou, China). Sequences are listed in Table S1. A549 cells were transfected with SiDR5 (100 nM), Scramble (100 nM) or transfection complex without any siRNA (Mock), respectively, using the Lipofectamine 2000 reagents (Invitrogen, Carlsbad, CA, USA) according to the manufacturer’s instructions. Transfections were repeated three times. Transfected cells were cultured for 18 h, then harvested for DR5 staining using PE mouse anti-human DR5 antibody or PE mouse IgG isotype as control, followed by FCS analysis for DR5 expression levels. Or transfected cells were treated for 24 h with vehicle, 1.0 ng/mL EV-T or/and 15 nM dinaciclib, respectively, followed by cell viability assay by CCK-8 Kit.

### 4.11. Detection of Cell Surface Expression of DR4 and DR5

Untreated cells were seeded and cultured 24 h before detection or seeded cells were allowed to settle down for overnight followed by treatments with therapeutic agents for 24 h and then staining for detection. Cells were stained in detergent-free buffer with PE mouse anti-human DR4 or anti-DR5 antibody or with PE mouse IgG as isotype control (Invitrogen), followed by flow cytometry assay to measure cell surface DR4 and DR5 expression levels. 

### 4.12. Mitochondrial Membrane Potential and F-actin Changes Observed by LCSM

The mitochondrion-specific dye JC-1 (Beyotime Biotechnology, Shanghai China) and FITC-phalloidin were used to detect changes in the mitochondrial membrane potential and cytoskeleton of tumor cells [56], respectively. Firstly, cells were cultured in laser scanning confocal dishes at a density of 2 × 10^5^ cells per well overnight. Then, cells in the experimental groups were treated with vehicle, 1.0 ng/mL EV-T or/and 15 nM dinaciclib for 24 h at 37 °C. JC-1 working solution was prepared according to the manufacturer’s protocols. Treated cells were washed with PBS, then stained with JC-1 for 20 min at 37 °C. Then, the samples were rinsed with JC-1-free staining buffer twice. For cytoskeleton staining, the cells were incubated with FITC–phalloidin for 1 h at 37 °C in dark and then washed twice with PBS. Finally, the stained samples were observed and imaged with a laser scanning confocal microscope (LSM800, Zeiss).

### 4.13. Western Blotting Analysis 

For immunoblotting detection of protein expression, cells or EVs were harvested and lysed for 30 min at 4 °C in radio-immunoprecipitation assay (RIPA) buffer (phosphate-buffered saline, 1% Igepal Ca-630, 0.5% sodium deoxycholate and 0.1% sodium dodecyl sulfate (Sigma, St. Louis, MO, USA) supplemented with complete protease inhibitor cocktail (Complete-mini; Roche Diagnostics GmbH, Mannheim, Germany). Protein concentration was determined by BCA assay (Beyotime Biotech). 10 µg of cellular or EV protein were prepared and resolved on 12% polyacrylamide sodium dodecyl sulfate (SDS-PAGE) gels, transferred to polyvinylidence fluoride (PVDF) membranes (Roche Diagnostics), blocked with 5% milk for one hour at room temperature (25 °C) and then incubated with primary antibodies at suggested dilution concentration at 4 °C for overnight. The used primary antibodies for α-tubulin (66031-I-Ig), XIAP (10037-I-Ig), CDK1 (10762-I-Ap), and TRAIL (66756-I-Ig) (dilution 1:1000) were all purchased from Proteintech (Chicago, IL, USA) and antibodies against human CDK9 (Ab76320), cFLIP (Ab8421), Mcl-1 (Ab32087), Bcl-2(Ab182858) and Bax (Ab182733) (dilution 1:2000) were from Abcam and anti-human CD63 was from Invitrogen (10628D). Second antibodies are anti-mouse or anti-rabbit antibodies conjugated with horseradish peroxidase (dilution 1:2000, Proteintech, Chicago, IL, USA). Immunoblots on the membrane was detected by an enhanced chemiluminescence method and photographed by BioImage Lab (Bio-Rad, Hercules, CA, USA). Western blotting bands were analyzed using the ImageJ software (National Institutes of Health, Bethesda, MA, USA) and normalized with α-tubulin as an internal loading control.

### 4.14. In Vivo Study

Female Balb/c nude mice (3–5 weeks old) were purchased from SPF Biotechnology Company (Beijing, China). All experimental studies were approved by the Animal Ethics Committee of South China University of Technology (Approval ID: 20191612135; Date: 21 June 2019). Animals were housed in pathogen-free conditions with filtered air, and autoclaved food and water were available ad libitum. Five million A549 cells were injected for each mouse in a total of 100 µL suspension, subcutaneously in the right flank with an insulin syringe. Tumors were measured every 2 days with Vernier caliper, and the tumor volume (TV) was calculated as TV (mm^3^) = d^2^ × D/2, where d and D are the shortest and the longest diameter, respectively. Animal body weight and general condition were also monitored and recorded. Mice were randomized into five groups: control, EV-T, dinaciclib (Dina), EV-T + Dina (Combi) and rTRAIL+Dina. The treatment schedule was composed of 3 injections, every 48 hours, with 50 μg per injection of EV-T (corresponding to 4.5 ng pure TRAIL), 160 μg dinaciclib (8 mg/kg animal weight), combination of EV-T or rTRAIL (4.5 ng) with dinaciclib (160 μg), or saline. Treatments started at 100 mm^3^ tumor volume (TV) with intratumor injections. The antitumor effect was assessed as TV inhibition percentage (TVI %) in treated versus control mice group. The tested EV dosage is far lower than that previously examined well tolerated dosage at 200 μg EVs per injection in vivo [11]. The experiment was carried out for two times using 5 animals per group. Mice were sacrificed 24 h after the last treatment or at the endpoint when first signs of animal distress examined, and mice tumor lesions and organs were collected.

### 4.15. Immuno Histochemistry (IHC)

Sections of formalin-fixed, paraffin-embedded tumor specimens were deparaffinized in xylene and hydrated in a graded alcohol series and then immersed the slides in sodium citrate antigen retrieval solution for antigen retrieval at 100 °C for 20 min. Endogenous peroxidase was blocked using 3% hydrogen peroxide in distilled water for 15 min. The tissue slides were incubated with 3% BSA solution (Servicebio, Beijing China) for 15 min at room temperature. Formalin-fixed and paraffin-embedded tissue sections (3–4 mm) were incubated with the following Abs: Ki67, CD20, CD11b, F4/80 (Servicebio) and cleaved-caspase-3 (Cell Signaling Technology, Danvers, MA, USA). UltraVisionQuanto Detection System HRP (Thermo Fisher Scientific Inc.,Waltham, MA, USA) and DAB (Liquid DABþ Substrate Chromogen System, Dako, Copenhagen, Denmark) were used to develop the reaction. TUNEL staining (ApopTag Peroxidase In Situ Apoptosis Detection Kit; Merck Millipore) was performed according to the manufacturer’s instructions. Images were acquired by AperioScanScope XT systems (Aperio Technologies, Leica Microsystems Srl, Wetzlar, Germany) or Eclipse E600 microscope (Nikon, Tokyo, Japan).

### 4.16. Statistical Analysis

Data were analyzed using GraphPad Prism 5.0 Software (GraphPad Software Inc., La Jolla, CA, USA) or Origin 7.5 software (OriginLab, Northampton, MA, USA). Differences between groups were analyzed by using Student’s t-test or Bonferroni multiple comparison statistic test. Significant probability values are denoted as * *p* < 0.05, ** *p* < 0.01, *** *p* < 0.001.

## 5. Conclusions

TRAIL can be secreted via EV by cells expressing the ligand. EV carried TRAIL (EV-T) can be combined with dinaciclib to markedly induce apoptosis in resistant cancer cells that express good levels of DR5. Combination therapy with EV-T and dinaciclib is a novel strategy for effective and safe cancer treatment.

## Supplementary Materials

The following are available online at https://www.mdpi.com/2072-6694/12/5/1157/s1, Figure S1: Whole immuno blotting for expression of cellular TRAIL (A), alpha-tubulin (B), EV-derived TRAIL (C) and EV marker CD 63 (D) with protein molecular weight ladder showing bands from 15 kDa to 70 kDa on the left, Figure S2: EV-T is more efficient for cancer cell killing than rTRAIL, Figure S3: Flow cytometry analysis of the cell surface expression of DR4 and DR5 in A549 cells. Figure S4: Whole immuno blotting for expression of CDK1, CDK9, cFLIP, XIAP, Mcl-1, Bcl-2, Bax and alalpha-tubulin with protein molecular weight ladder showing bands from 15 kDa to 70 kDa on the left, Figure S5: EV-T is more efficient than soluble rTRAIL for inhibition and killing of A549 subcutaneous xenograft tumor when combined with dinaciclib, Figure S6: Changes in animal body weight. Figure S7: H&E staining of mice organs. Table S1: Sequences of 3 duplex siRNAs against DR5 and 1 duplex scramble siRNA.
