# Supplementary material for: Extracellular Vesicle Delivery of TRAIL Eradicates Resistant Tumor Growth in Combination with CDK Inhibition by Dinaciclib
Source: Cancers (Basel). 2020 May 4;12(5):1157. doi: 10.3390/cancers12051157 (PMC7281120; doi:10.3390/cancers12051157)
Supplement: Supplementary file 1 [file cancers-12-01157-s001.pdf]

# Supplementary Materials: Extracellular Vesicle Delivery of TRAIL Eradicates Resistant Tumor Growth in Combination with CDK Inhibition by Dinaciclib

**Changhong Ke, Huan Hou, Jiayu Li, Kui Su, Chaohong Huang, Yue Lin, Zhiqiang Lu, Zhiyun Du, Wen Tan and Zhengqiang Yuan**

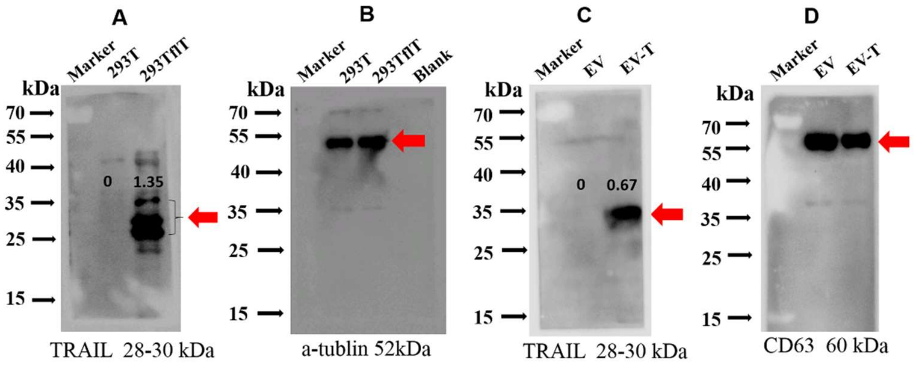


**Figure S1.** Whole immuno blotting for expression of cellular TRAIL (**A**), alpha-tubulin (**B**), EV-derived TRAIL(**C**) and EV marker CD 63 (**D**) with protein molecular weight ladder showing bands from 15 kDa to 70 kDa on the left. The values above the target bands are densitometry reading/intensity ratio of each band relative to alalpha-tubulin in cellular lysates and to CD63 in EVs, respectively.2.2. EV-T showed specific cytotoxicity to the highly TRAIL-resistant A549 line.

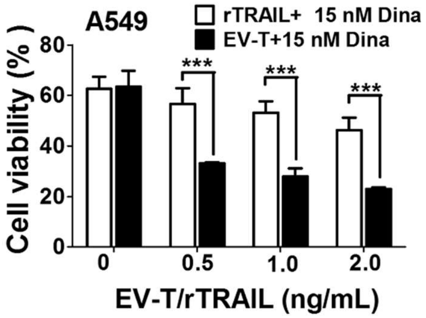


**Figure S2.** EV-T is more efficient for cancer cell killing than rTRAIL. The TRAIL-resistant A549 line was co-treated with 15nM dinaciclib (Dina) and vehicle (0), 0.5, 1.0, 2.0 ng/mL of rTRAIL or EV-T for 24 h, respectively, followed by cell proliferation and viability assessment by CCK-8 kit. Values are mean ± S.E.M ($n$ = 3).

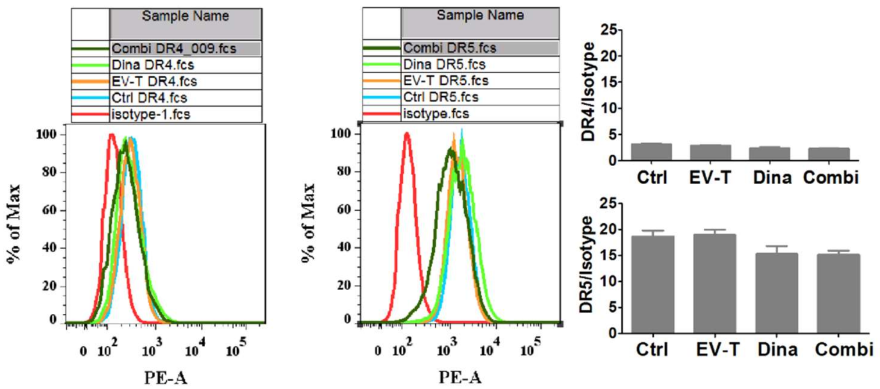


**Figure S3.** Flow cytometry analysis of the cell surface expression of DR4 and DR5 in A549 cells. Cells were treated with saline (Ctrl), EV-T at 1.0 ng/mL (EV-T) or/and dinaciclib at 15 nM (Dina, Combi) for 24 h, respectively, followed by immuno fluorescent staining with PE mouse Abs against human IgG (isotype), DR4 and DR5, respectively, analyzed by FACS. Median PE fluorescence intensity (MFI) of samples relative to isotype labeling was used to assess expression levels of DR4 and DR5. Data represent mean ± S.E.M ($n$ = 4).

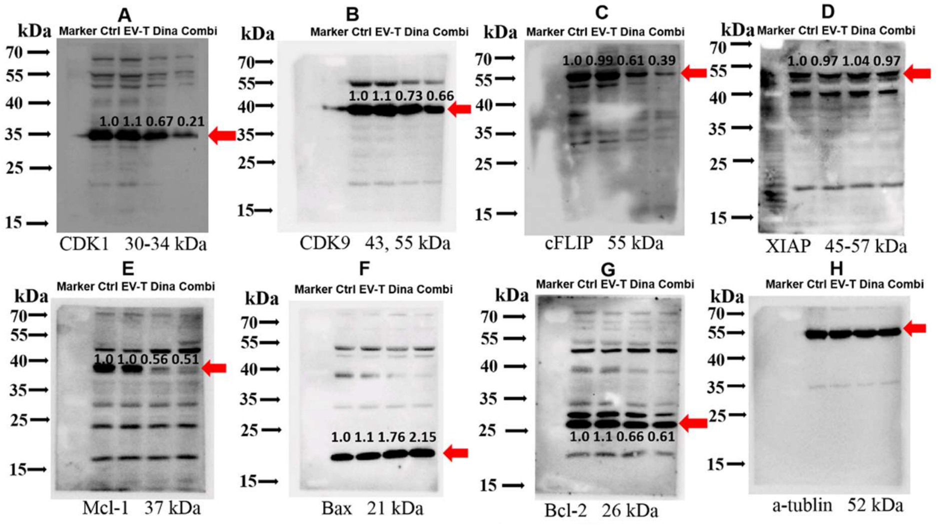


**Figure S4.** Whole immuno blotting for expression of CDK1, CDK9, cFLIP, XIAP, Mcl-1, Bcl-2, Bax and alalpha-tubulin with protein molecular weight ladder showing bands from 15 kDa to 70 kDa on the left. The sampling order of all the proteins from left to right is marker, vehicle (Ctrl), 1.0 ng/mL of EV-T (EV-T) or/and 15 nM of dinaciclib (Dina) (Combi). The values above the target bands are densitometry reading/intensity ratio of each band relative to alalpha-tubulin respectively. Expression is shown relative to Ctrl, the value for which was set to 1.

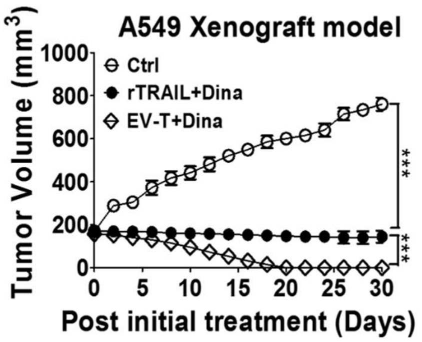


**Figure S5.** EV-T is more efficient than soluble rTRAIL for inhibition and killing of A549 subcutaneous xenograft tumor when combined with dinaciclib. Experimental treatment schedule is same with that shown in Figure 7A. Each treatment comprises of intratumoral injection per animal of saline vehicle (Ctrl), 4.5 ng rTRAIL+ 160 μg dinaciclib (rTRAIL+Dina), or 4.5 ng EV-T+ 160 μg dinaciclib (EV-T+Dina), total three injections were performed with a 48-h interval for each animal. Tumor growth/volume curves were plotted along with days post initial treatment to reflect treatment effects. All values are mean ± SD ($n$ = 5), *** $p < 0.001$, Students' T test.

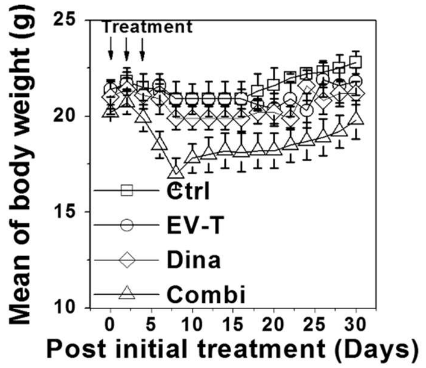


**Figure S6.** Changes in animal body weight. In vivo tested mice were monitored for body weight change during the treatment period. Animal weight was measured once for each mouse every two days over 30 days, and the mean value of each treatment group was plotted against treatment time until the endpoint. All values are mean ± S.E.M ($n$ = 5).

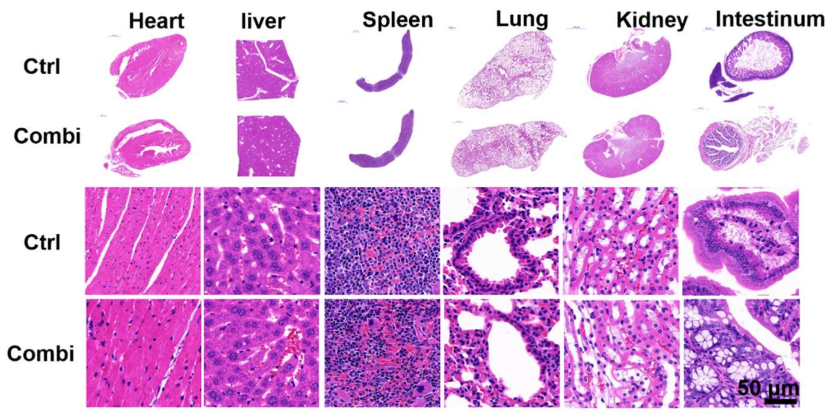


**Figure S7. H&E staining of mice organs.** Animal organs including heart, lung, liver, spleen, kidney and intestine were collected and stained by H&E to check any sign of adverse side effects at the experimental endpoints from mice treated by saline (Ctrl) or combination of EV-T and dinaciclib

(Combi) as described in Figure 7B. The upper panel illustrates gross view of tissue sections while the lower panel shows the microscopic view.

**Table s1.** Sequences of 3 duplex siRNAs against DR5 and 1 duplex scramble siRNA.

| SiRNA | Sequence（5′-3′） |
|---|---|
| 1-SiDR5-sense | CAGCCGUAGUCUUGAUUGUdTdT |
| 1-siDR5-anti sense | ACAAUCAAGAUCACGGCUGdTdT |
| 2-SiDR5-sense | GACAGGACUAUAGCACUCAdTdT |
| 2-siDR5-anti sense | UGAGUGCUAUAGUCCUGUCdTdT |
| 3-SiDR5-sense | CAAGGUCGGUGAUUGUACAdTdT |
| 3-siDR5-anti sense | UGUACAAUCACCGACCUUGdTdT |
| Scramble-sense | UUCUCCGAACGUGUCACGUdTdT |
| Scramble-anti sense | ACGUGACACGUUCGGAGAAdTdT |
